# Comment on “Anion Gap Toxicity in Alloxan Induced Type 2 Diabetic Rats Treated with Antidiabetic Noncytotoxic Bioactive Compounds of Ethanolic Extract of *Moringa oleifera*”

**DOI:** 10.1155/2019/3930587

**Published:** 2019-11-20

**Authors:** Javad Beheshtipour

**Affiliations:** Young Researchers and Elite Club, Sanandaj Branch, Islamic Azad University, Sanandaj, Iran

I read with interest the article by Omabe et al., entitled “Anion Gap Toxicity in Alloxan Induced Type 2 Diabetic Rats Treated with Antidiabetic Noncytotoxic Bioactive Compounds of Ethanolic Extract of *Moringa oleifera,*” published in this journal (vol. 2014, Article ID 406242) [1]. It is interesting to note that this scientific subject has been dealt with, but one point in the article is the focus of my attention which is discussed below.

Alloxan is one of the oldest chemicals to induce experimental diabetes. It induces type 1 diabetes through a single dose administration in laboratory animals. Alloxan acts in two ways: it selectively inhibits glucose-induced insulin secretion through specific inhibition of glucokinase, the glucose sensor of the beta cell, and it causes a state of insulin-dependent diabetes through its ability to induce reactive oxygen species (ROS) formation, resulting in the selective necrosis of pancreatic beta cells [2, 3]. Therefore, alloxan cannot induce type 2 diabetes.

In the article by Omabe et al., type 2 diabetes has been induced by a single intraperitoneal injection of alloxan, which is not correct due to its mechanism of action.
